# Tumor cell invasion of collagen matrices requires coordinate lipid agonist-induced G-protein and membrane-type matrix metalloproteinase-1-dependent signaling

**DOI:** 10.1186/1476-4598-5-69

**Published:** 2006-12-08

**Authors:** Kevin E Fisher, Andreia Pop, Wonshill Koh, Nicholas J Anthis, W Brian Saunders, George E Davis

**Affiliations:** 1 Department of Pathology and Laboratory Medicine, Texas A&M University System Health Science Center, College Station, TX 77843, USA; 2 Department of Medical Pharmacology and Physiology, University of Missouri School of Medicine, Columbia, MO 65212, USA; 3 Department of Pathology and Anatomical Sciences, University of Missouri School of Medicine, Columbia, MO 65212, USA

## Abstract

**Background:**

Lysophosphatidic acid (LPA) and sphingosine 1-phosphate (S1P) are bioactive lipid signaling molecules implicated in tumor dissemination. Membrane-type matrix metalloproteinase 1 (MT1-MMP) is a membrane-tethered collagenase thought to be involved in tumor invasion via extracellular matrix degradation. In this study, we investigated the molecular requirements for LPA- and S1P-regulated tumor cell migration in two dimensions (2D) and invasion of three-dimensional (3D) collagen matrices and, in particular, evaluated the role of MT1-MMP in this process.

**Results:**

LPA stimulated while S1P inhibited migration of most tumor lines in Boyden chamber assays. Conversely, HT1080 fibrosarcoma cells migrated in response to both lipids. HT1080 cells also markedly invaded 3D collagen matrices (~700 μm over 48 hours) in response to either lipid. siRNA targeting of LPA_1 _and Rac1, or S1P_1_, Rac1, and Cdc42 specifically inhibited LPA- or S1P-induced HT1080 invasion, respectively. Analysis of LPA-induced HT1080 motility on 2D substrates vs. 3D matrices revealed that synthetic MMP inhibitors markedly reduced the distance (~125 μm vs. ~45 μm) and velocity of invasion (~0.09 μm/min vs. ~0.03 μm/min) only when cells navigated 3D matrices signifying a role for MMPs exclusively in invasion. Additionally, tissue inhibitors of metalloproteinases (TIMPs)-2, -3, and -4, but not TIMP-1, blocked lipid agonist-induced invasion indicating a role for membrane-type (MT)-MMPs. Furthermore, MT1-MMP expression in several tumor lines directly correlated with LPA-induced invasion. HEK293s, which neither express MT1-MMP nor invade in the presence of LPA, were transfected with MT1-MMP cDNA, and subsequently invaded in response to LPA. When HT1080 cells were seeded on top of or within collagen matrices, siRNA targeting of MT1-MMP, but not other MMPs, inhibited lipid agonist-induced invasion establishing a requisite role for MT1-MMP in this process.

**Conclusion:**

LPA is a fundamental regulator of MT1-MMP-dependent tumor cell invasion of 3D collagen matrices. In contrast, S1P appears to act as an inhibitory stimulus in most cases, while stimulating only select tumor lines. MT1-MMP is required only when tumor cells navigate 3D barriers and not when cells migrate on 2D substrata. We demonstrate that tumor cells require coordinate regulation of LPA/S1P receptors and Rho GTPases to migrate, and additionally, require MT1-MMP in order to invade collagen matrices during neoplastic progression.

## Background

Tumor cell invasion is a complex process involving genetic and cellular alterations which lead to proteolysis and dispersion through three-dimensional biological barriers [1-4]. Type I collagen is the most abundant component of the extracellular matrix (ECM), and is therefore a significant obstacle for tumor cell dissemination into the lymphatics, vasculature, and surrounding areas [5,6]. Thus, in most cases, collagen must be degraded in order for tumor cells to spread into surrounding anatomic structures and metastasize [7]. Cell migration, governed by polarity and reorganization of the cellular cytoskeleton, is also an integral aspect of tumor cell invasion [8,9]. Dissecting the molecular requirements of tumor cell migration and invasion is necessary because the latter, in conjunction with metastasis, is a significant cause of morbidity and mortality in cancer patients [10].

Recent reports have identified two lipid signaling molecules, lysophosphatidic acid (LPA) and sphingosine 1-phosphate (S1P), in many critical biological events such as development, angiogenesis, inflammation, and wound repair [11-15]. LPA and S1P function as extracellular lipid agonists which activate a subfamily of G protein-coupled receptors (GPCRs) and subsequent downstream effectors such as the small GTPases RhoA, Rac1, and Cdc42 [16-20]. In addition to fundamental cellular signaling, LPA, particularly in ovarian cancer, and S1P have been implicated in tumor cell proliferation, anti-apoptosis, cytoskeletal rearrangement and migration, and invasion [21-26]. LPA_1–3 _receptors are thought to be involved in cell motility and are aberrantly expressed in cancer cells [25,27]. S1P_1–3 _are also involved in the regulation of cell migration and play important roles in the vascular system [11,15].

Additional reports have linked LPA and S1P to the matrix metalloproteinases (MMPs) [24,28,29]. The role of MMPs in tumor invasion has been well documented [30-33] and clinical cancer therapeutic trials have sought to target these molecules albeit with disappointing results [34,35]. Membrane-type matrix metalloproteinase 1 (MT1-MMP), also known as MMP-14, is a membrane-bound collagenase that has been shown to localize to the leading edge of invading cells, degrade surrounding extracellular matrix, and play a pivotal role in cancer cell dissemination [36-39]. The objective of the current study, therefore, is to further characterize the roles of LPA, S1P, and MMPs (specifically MT1-MMP) in the processes of tumor cell migration and invasion using both 2D migration analysis and 3D type I collagen invasion assays.

Our data demonstrate that LPA stimulated and S1P inhibited migration of most tumor lines tested. In contrast, HT1080 fibrosarcoma cells migrated in response to both lipids. Invasion of 3D collagen matrices of HT1080 cells, but not migration in either Boyden chambers or on collagen-coated plastic, was blocked in the presence of synthetic MMP inhibitors, and TIMP-2, -3, or -4. This indicates that lipid-induced invasion of 3D collagen matrices is an MT-MMP-dependent event. Transfection of HT1080 cells with siRNAs identified LPA_1_, S1P_1_, Rac1, Cdc42, and MT1-MMP as key components of the invasion response. Additional experiments revealed that SKOV3 and HEK293 cell lines, which express low levels of MT1-MMP, do not invade LPA-containing 3D collagen matrices despite a marked ability to undergo LPA-induced migration. When HEK293 were transfected with cDNA encoding MT1-MMP, LPA-induced invasion of 3D collagen matrices occurred. Our results demonstrate that lipid agonist-induced tumor cell invasion requires MT1-MMP, and its signaling occurs via LPA_1_, S1P_1_, Rac1, and Cdc42. We propose that coordinated signaling events between LPA and S1P receptors, their effectors, and MT1-MMP are required during tumor cell invasion of 3D collagen matrices, but MT1-MMP is not required for tumor motility on 2D collagen substrates.

## Results

### LPA stimulates while S1P either inhibits or stimulates migration of tumor cells

**Figure 1:**
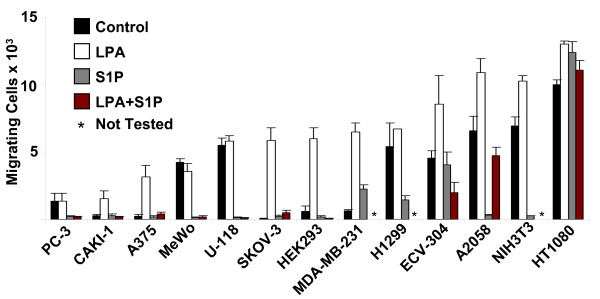
**LPA stimulates and S1P inhibits migration of most tumor cell lines**. Tumor cell migration analysis was performed on 8 μm polycarbonate gelatin-coated membranes and 20 μg/ml of fibronectin. LPA and/or S1P (1 μM) were added to the lower chambers and cells were allowed to migrate for 4 hours. Membranes were removed, stained, and migrating cells were quantitated using Scion^® ^software and Microsoft Excel^®^. Data are expressed as mean number of migrating cells × 10^3 ^(± S.D.) and represent the results of quadruplicate experiments.

To study the effects of LPA and S1P on migration of tumor cell lines, modified Boyden chambers were utilized. Cells were allowed to migrate for 4 hours in the presence or absence of LPA or S1P. LPA stimulated migration in all tumor cell lines examined, while S1P blocked migration in almost all lines (Fig. 1). These data identified LPA as the most potent migratory stimulus for the tumor cell lines evaluated compared to chemotactic growth factors such as EGF, FGF-2, HB-EGF, IGF-1, and PDGF (data not shown). Thus, in this study we focused on LPA as a stimulus for tumor cell migration and invasion.

Four cell lines were selected for further study due to their marked LPA-migration response namely A2058 melanoma, SKOV3 ovarian carcinoma, human embryonic kidney (HEK) 293, and HT1080 fibrosarcoma. We have previously shown that S1P strongly influences invasive behavior of human endothelial cells (another highly invasive cell type) in 3D collagen matrices [28,40]. The effects of S1P were therefore also evaluated in these cell lines. LPA produced a strong migratory response for these cell lines, and in all but one case (HT1080) S1P inhibited migration. Interestingly, HT1080 cells exhibited a strong migratory response in the presence of both LPA and S1P, contrary to the trend of the other cell lines. This result is particularly intriguing because HT1080 cells are among the most invasive tumor cell lines in both *in vivo *and *in vitro *settings [41,42], suggesting a role for S1P responsiveness in highly invasive tumor cells.

### Tumor cell invasion of 3D collagen matrices mimics the lipid agonist migration profile in select cell lines

**Figure 2:**
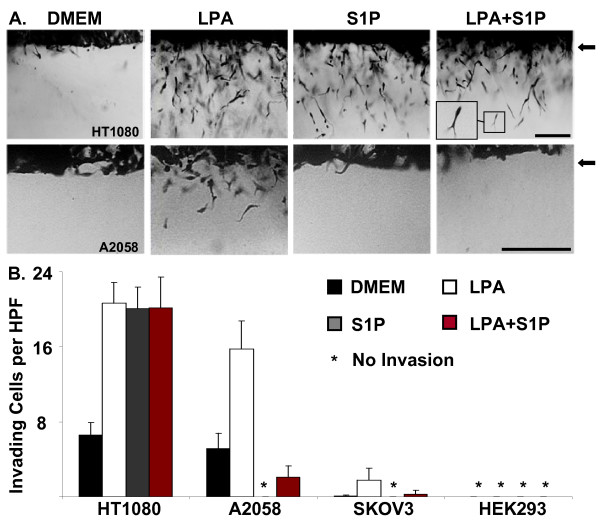
**LPA and S1P regulate tumor cell invasion of 3D collagen matrices**. (A) HT1080, A2058, SKOV3 or HEK293 cells were induced to invade 3D collagen gels (3.75 mg/ml or 2.0 mg/ml) under serum-free conditions in the presence or absence of LPA alone (1 μM), S1P alone (1 μM), or both LPA and S1P (1 μM each). After 48 hours, cultures were fixed, stained, and images were acquired to demonstrate the effects of LPA and S1P on tumor cell invasion. Arrowheads indicate the position of the tumor cell monolayer at the initiation of invasion. Magnification = 20×. Scale bar = 50 μm. Inset (panel A) = higher power magnification of invading HT1080 cell. (B) Quantitation of tumor invasion from panel A. Data are expressed as mean numbers of invading cells per HPF (20×) (± S.D.) from a minimum of 20 fields.

An *in vitro *A/2 96-well invasion assay was utilized to study the effects of LPA and S1P on tumor cell invasion of highly concentrated (3.75 mg/ml or 2.0 mg/ml) 3D type I collagen gels. LPA or S1P were polymerized in collagen gels at 1 μM, and the four selected cell lines were placed on the surface of either 3.75 or 2.0 mg/ml collagen matrices and allowed to invade for 48 hours. Cultures were then fixed and stained for imaging and quantification as shown in Fig. 2A and 2B. Invading cultures are presented as cross-sections to allow visualization of the monolayer (arrows) and the underlying collagen matrix containing invading tumor cells. The HT1080 cell line invaded and migrated dramatically in response to both LPA and S1P (Fig. 1 and Fig. 2). Invasion of the A2058 cell line was stimulated by LPA, but was inhibited by S1P, consistent with the migration profile of this cell line (Fig. 1 and 2A). SKOV3 and HEK293 cells markedly migrated in response to LPA in migration assays (Fig. 1), but were unable to invade 3D collagen matrices in response to these lipids (Fig. 2B).

### Time-lapse analysis of LPA and S1P-induced tumor invasion of collagen matrices

**Figure 3:**
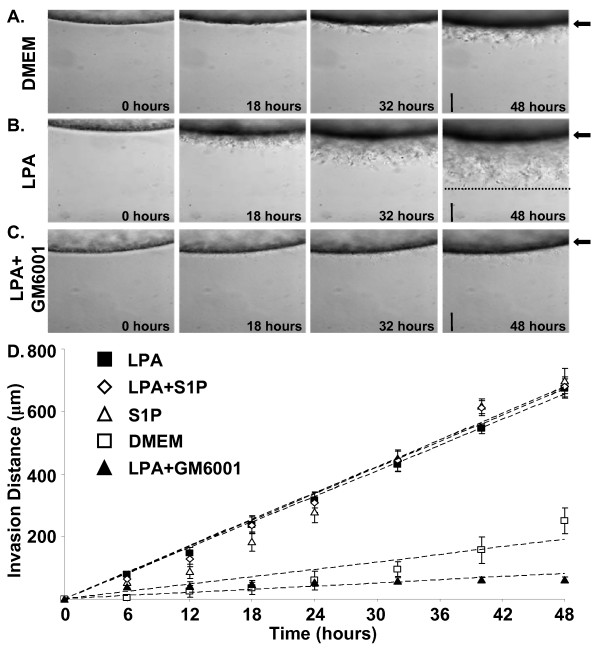
**Time-lapse analysis of HT1080 invasion: LPA and S1P induce ~700 μm of invasion of 3D collagen matrices**. (A) HT1080 cells were seeded onto 3.75 mg/ml collagen gels in glass casings (see methods for details) and allowed to invade for 48 hours in the presence of DMEM, LPA, S1P, LPA and S1P (1 μM each) or LPA (1 μM) + GM6001 (5 μM). Digital images of representative fields were captured at ten minute intervals. Using Metamorph^®^, invasion distance measurements at each time point from triplicate wells were obtained (n = 15). Magnification = 10×. Scale bars = 200 μm. (B) Data from panel A were plotted versus time and rates of invasion were calculated using linear regression (see Table 1). Data are expressed as mean invasion distance (n = 3 wells, ± S.D.) for each time point from triplicate experiments.

Additional experiments utilized a new method for time-lapse analysis of tumor cell invasion. In this assay, collagen gels were placed into thin walled square glass casings. Tumor cells were then seeded onto the surface of the gel and the casings were placed horizontally so that tumor invasion could be imaged parallel to the surface of the gel and monolayer. Invasion time course experiments revealed that LPA or S1P significantly increased the rate of invasion of HT1080 cells as compared to DMEM control (Fig. 3A, B, and 3D and Table 1). When 3.75 mg/ml type I collagen gels containing either LPA or S1P were used, HT1080 cells invade ~700 μm in 48 hours at a rate of 14–15 μm/hr (See additional file 1: LPA Invasion to view 24 hours of the time-lapse imaging). Notably, the addition of a synthetic inhibitor of MMPs (GM6001) completely blocked LPA-induced invasion of 3D collagen matrices over this time period (Fig. 3C, D and Table 1; see additional file 2: LPA+GM6001 Invasion to view 24 hours of the time-lapse imaging) establishing a requirement for MMPs in this process (see later on).

**Table 1:** LPA and S1P accelerate MT1-MMP-mediated HT1080 invasion in 3D collagen matrices

**Condition**	**Rate of Invasion (μm/hr)**	**R**^**2**^
DMEM	4.930	0.8977
LPA	13.971	0.9986
S1P	15.672	0.9777
LPA + S1P	14.941	0.9916
LPA + GM6001	1.010	0.6971
Luciferase*	13.873	0.9997
Scrambled MT1-MMP*	13.532	0.9988
si MT1-MMP*	5.366	0.9584

HT1080 cells were allowed to invade for 48 hours in the presence or absence of the specified lipid. siRNA treated HT1080 cells (Luciferase, MT1-MMP, or a control scrambled MT1-MMP) invaded in the presence of 1 μM LPA. Invasion distances were obtained with Metamorph^® ^software by measuring pixel distance at each time point (five measurements per time point of duplicate or triplicate experiments.) Rates of invasion were calculated using linear regression analysis. R^2 ^values denote the predictive potential of distance given time where 1.0 equals an exact correlation (* denotes separate experiment).

### S1P and LPA stimulate HT1080 invasion of 3D type I collagen matrices through S1P_1_, LPA_1_, Rac1, and Cdc42

**Figure 4:**
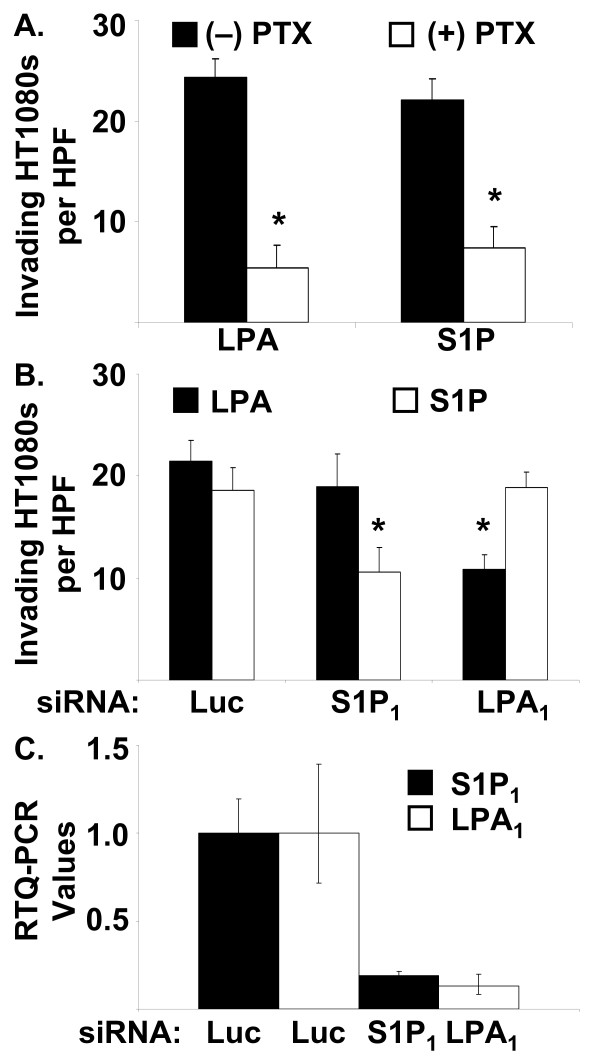
**LPA_**1 **_and S1P_**1 **_regulate HT1080 invasion of 3D collagen matrices in response to LPA and S1P, respectively**. (A) HT1080 cells were allowed to invade in the presence of either LPA or S1P (1 μM) in the presence (+) or absence (-) of 100 ng/μl of pertussis toxin (PTX). Cells were fixed, stained, and quantitated as described in Fig. 2. (B) HT1080 cells were transfected with siRNAs targeting the indicated genes and allowed to invade in the presence of either LPA or S1P (1 μM). Data are reported as mean numbers of invading cells per HPF (20×) (± S.D.) from a minimum of 20 fields. Statistical significance of means was determined using t-test where * = p < 0.01 relative to Luciferase (Luc) control for each lipid. (C) Real-time Quantitative PCR (RTQ-PCR) values for *LPA*_*1 *_and *S1P*_*1 *_mRNA normalized against *18S *RNA were used to illustrate selective targeting of the designated siRNA.

LPA and S1P have been shown to bind and signal through GPCRs [12,18,27]. Therefore, we elected to characterize which receptors were involved in lipid-induced invasion. Treatment of HT1080 cells with 100 ng/μl of pertussis toxin (PTX) blocked invasion (Fig. 4A) suggesting a role for GPCRs in this process, particularly those that signal through G_αi _[43,44]. To identify the receptors required for invasion, siRNAs targeting LPA_1–3 _and S1P_1–3 _receptors were used. Interestingly, when HT1080 cells were allowed to invade in the presence of LPA, invasion was blocked only by siRNAs targeting LPA_1 _(Fig. 4B and data not shown). When HT1080 cells were allowed to invade in the presence of S1P, invasion was blocked by siRNAs targeting only S1P_1 _(Fig. 4B and data not shown). Efficiency and specificity of mRNA knockdown was confirmed by Real-Time Quantitative PCR analysis (Fig. 4C). These data suggest that LPA- and S1P-induced invasion signal through different receptors and are thus dissociable processes. These data are consistent with the observed inhibition of invasion by PTX.

**Figure 5:**
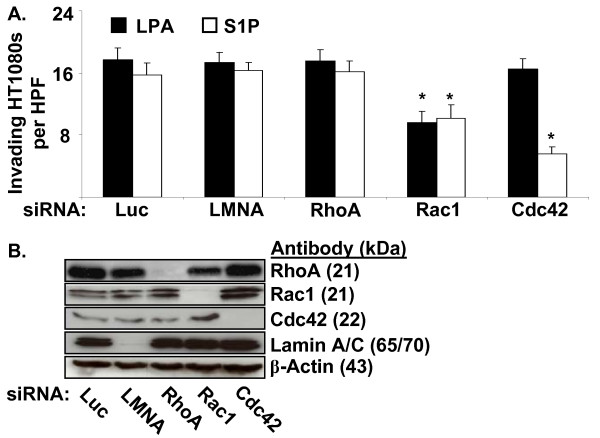
**Rac1 and Cdc42 are downstream effectors involved in LPA- and S1P-induced HT1080 invasion of 3D collagen matrices**. (A) HT1080 cells were transfected with siRNAs targeting the genes indicated and allowed to invade in the presence either LPA or S1P (1 μM). Data are expressed as mean numbers of invading cells per HPF (20×) (± S.D.) from a minimum of 20 fields. Statistical significance of means was determined using t-test where * = p < 0.01 relative to Luciferase (Luc) control for each lipid. LMNA = Lamin A/C. (B) siRNA transfected HT1080 cell lysates were prepared for Western blot analysis. Lysates were probed for Lamin A/C, RhoA, Rac1, and Cdc42 to demonstrate siRNA specificity and probed for Actin as a loading control.

We then investigated which of the GPCR effectors were involved in lipid-induced invasion in our system. RhoA, Rac1, and Cdc42 have each been implicated in tumor motility and invasion [45,46], are downstream effectors of both LPA_1 _and S1P_1 _[47,48], and therefore were candidates for targeting. We transfected HT1080 cells with siRNAs targeting either RhoA, Rac1, Cdc42, or control siRNAs (Luciferase and Lamin A/C) and assessed invasion in the presence of 1 μM LPA or S1P. siRNA targeting of RhoA had no effect on invasion in the presence of either lipid (Fig. 5A). siRNA targeting of Rac1 inhibited invasion in the presence of either LPA or S1P. Interestingly, siRNA targeting of Cdc42 only inhibited S1P-induced invasion (Fig. 5A). Efficiency and specificity of siRNA transfection was assessed by Western blotting (Fig. 5B). These data indicate that Rac1 and Cdc42 are important GPCRs involved in lipid-induced invasion in our system. Furthermore, Rac1 is involved in both LPA and S1P-induced invasion of 3D collagen matrices, while Cdc42 appears to be required only for S1P-induced invasion.

### MMPs are required for lipid agonist-induced tumor cell motility in 3D collagen matrices but not on 2D collagen substrates

**Figure 6:**
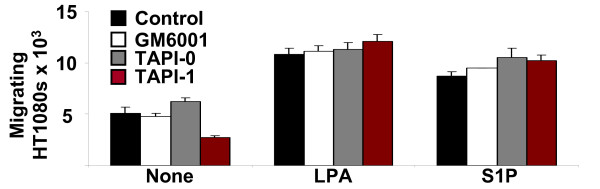
**HT1080 cell motility using modified Boyden chambers does not require MMPs**. HT1080 cells were seeded onto porous (8 μm) polycarbonate membranes coated with type I collagen (1 mg/ml) and allowed to migrate for four hours in the presence of 1 μM LPA or S1P in the presence or absence of synthetic MMP inhibitors (5 μM GM6001, 5 μM TAPI-0, or 5 μM TAPI-1). Membranes were removed, stained, and migrating cells were quantitated using Scion^® ^software and Microsoft Excel^®^. Data are expressed as mean number of migrating cells × 10^3 ^(± S.D.) and represent the results of quadruplicate experiments.

**Figure 7:**
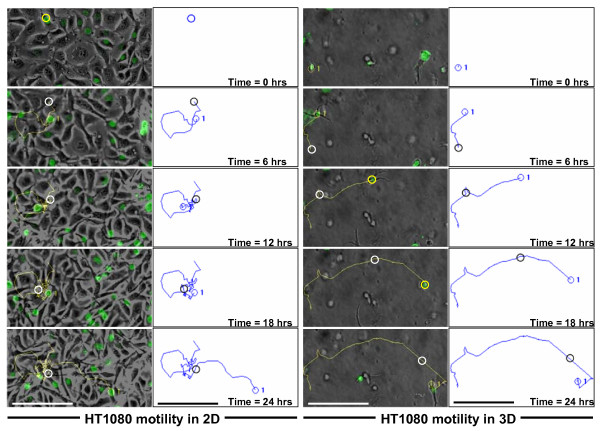
**Time-lapse assessment of lipid agonist-induced HT1080 cell motility on 2D collagen substrates versus 3D collagen matrices**. Nuc-GFP HT1080 cells were seeded on 2D plastic dishes coated with 50 μg/ml of collagen or embedded within a 3.75 mg/ml type I collagen matrix and their motility was analyzed using Metamorph^® ^software. Digital fluorescent images were acquired every 15 minutes and arranged in sequential order. A single cell is shown either moving on top of a dish (2D) or through 3.75 mg/ml collagen matrix (embedded in 3D) at 6 hour intervals for 24 hours. Tracings (generated by Metamorph^®^) of the continuous movement of these cells over the 24 hour time period are shown by the blue lines. (Note: Metamorph^® ^creates a yellow line on a black background which is difficult to see. Therefore, the images were inverted to turn the yellow line blue and the black background white.) A blue (yellow) circle indicates the current position of the cell and a black (white) circle indicates the previous position. Subsequent tracings were converted to grayscale. Magnification = 20×. Scale bars = 50 μm.

Previous reports have indicated that tumor cell motility in 3D matrices can occur in the absence of proteolysis (MMPs) [3,49], and that in some cases, MMPs are necessary for motility on 2D substrates [50,51]. Therefore using additional assays, we investigated whether lipid agonist-induced motility and/or invasion of 3D collagen matrices required MMPs. In modified Boyden chambers, the addition of the synthetic MMP inhibitors GM6001, Tumor-necrosis-factor-α protease inhibitor (TAPI)-0, and TAPI-1 had no effect on HT1080 migration (Fig. 6). To compare 2D vs. 3D motility, the lipid-induced motility of HT1080 cells stably expressing nuclear GFP (Nuc-GFP HT1080) in 2D or 3D was assessed (Fig. 7). Briefly, Nuc-GFP HT1080 cells were either seeded on top of plastic dishes coated with 50 μg/ml of collagen (2D) or embedded and polymerized within a 3.75 mg/ml collagen gel (3D). Cells were tracked using time-lapse fluorescence microscopy, and data was analyzed using Metamorph^®^software.

**Figure 8:**
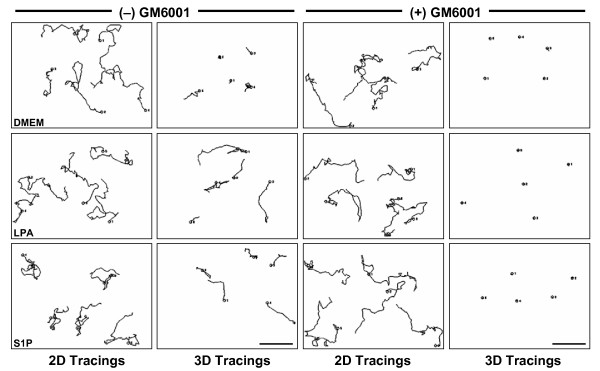
**Lipid agonist-induced tumor cell motility requires MMPs in 3D collagen matrices but not on 2D collagen substrates**. Five individual cell tracings generated by Metamorph^® ^(grayscale) in the presence of the specified lipid with or without 5 μM GM6001 (see Table 2 for distance and velocity calculations). Magnification = 20×. Scale bars = 100 μm.

Metamorph^® ^generated tracings (lines) by tracking nuclear movement of individual cells over 24 hours (see methods and figure legends for additional details). In 2D, the same distances (~300 μm) and velocities (~0.21 μm/min) of migration were noted regardless of the treatment (DMEM vs. LPA vs. S1P; One-way ANOVA: p = 0.767). The addition of GM6001 only slightly decreased (DMEM) or increased (S1P) the distances (~277 μm and ~322 μm) and velocities of migration (0.019 μm/min and 0.023 μm/min; One-way ANOVA: p < 0.01; Fig. 8 and Table 2; see additional file 3 and 4: 2D LPA and 2D LPA+GM6001 to view the time-lapse imaging of LPA-induced 2D motility with and without GM6001, respectively). However, when distances and velocities of cells embedded within a 3.75 mg/ml 3D collagen matrix were assessed, the lipid treatment and the addition of GM6001 had marked effects. In the presence of either LPA or S1P cells invaded ~50 μm further (~125 μm vs. ~75 μm – t-test p < 0.01 for both) and reached a velocity ~70% greater (0.085 μm/min vs. ~0.050 μm/min – t-test p < 0.01 for both; see Table 2) compared to invasion with no lipid (DMEM). In the presence of DMEM, LPA, or S1P with GM6001 added to the culture media, the distance (~45 μm) and velocity (0.030 μm/min) of the invading cells was markedly decreased (t-test p < 0.01) compared to treatment with DMEM or the lipid alone (Fig. 8; additional file 5 and 6: 3D LPA and 3D LPA+GM6001 to view the time-lapse imaging of LPA-induced invasion of cells embedded in 3D with and without GM6001, respectively). Together, these data demonstrate that lipid-induced invasion of 3D collagen matrices is a distinct process from lipid-induced migration through chemotactic chambers or on 2D substrata. In our assays, lipid-induced tumor cell invasion of 3D collagen barriers requires MMPs, whereas migration in 2D does not. Furthermore, these data demonstrate the marked ability of lipid agonists to induce tumor cell invasion of 3D collagen matrices, and that this invasion requires MMP activity.

**Table 2:** The synthetic MMP inhibitor GM6001 decreases distance and reduces velocity of lipid-induced HT1080 motility in 3D collagen matrices but not on 2D collagen substrates.

**Condition (over 24 hours)**	**Avg. Distance μm (2D)**	**Avg. Distance μm (3D)**	**Avg. Velocity μm/min (2D)**	**Avg. Velocity μm/min (3D)**
DMEM	303.68 ± 71.1	73.07 ± 31.74	0.21 ± 0.049	0.050 ± 0.022
DMEM + GM6001	277.38 ± 29.38	45.06 ± 15.17	0.19 ± 0.020	0.031 ± 0.010
LPA	298.58 ± 41.18	128.00 ± 56.98	0.21 ± 0.029	0.088 ± 0.039
LPA + GM6001	303.18 ± 55.41	40.43 ± 6.08	0.21 ± 0.038	0.028 ± 0.0042
S1P	303.05 ± 33.78	116.24 ± 25.39	0.21 ± 0.028	0.080 ± 0.017
S1P + GM6001	322.93 ± 69.05	44.598 ± 9.25	0.23 ± 0.049	0.031 ± 0.0057
Luciferase*	N/A	138.43 ± 57.71	N/A	0.096 ± 0.040
si MT1-MMP*	N/A	92.15 ± 35.90	N/A	0.064 ± 0.025

Nuc-GFP HT1080 cells were seeded on 2D plastic dishes coated with 50 μg/ml of collagen or embedded within a 3.75 mg/ml type I collagen matrix and their motility was analyzed using Metamorph^® ^software (see Fig. 7) in the presence of the specified lipid with or without GM6001. siRNA (Luciferase or custom MT1-MMP) treated nuc-GFP HT1080 cells were only analyzed in 3D (see Fig. 14). Data are expressed as the average total distance (μm) or average velocity (μm/min) ± S.D. of 15 tracings per condition. [Note: 96 distances and velocities (144 for siRNA experiments) were generated per tracing for a total of 1440 (2160) data points]. Statistical significance of means was determined using either One-way ANOVA or t-test where p < 0.05 was deemed statistically signficant (see text for specific comparisons).

### MT1-MMP is a key proteinase required for lipid-induced tumor cell invasion of 3D collagen matrices

**Figure 9:**
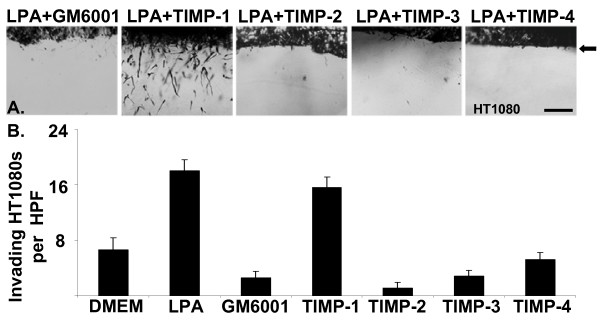
**MT-MMPs are required for tumor cells to invade 3D collagen matrices**. (A) HT1080 cells were induced to invade 3D collagen gels (3.75 mg/ml) as in Fig. 2 in the presence of 1 μM LPA and the synthetic MMP inhibitor GM6001 (5 μM), or TIMP-1, -2, -3, or -4 (5 μg/ml). Arrowheads indicate the position of the tumor cell monolayer at the initiation of invasion. Magnification = 20×. Scale bar = 50 μm. (B) Quantitation of HT1080 invasion from panel A. Data are expressed as mean numbers of invading cells per HPF (20×) (± S.D.) from a minimum of 20 fields.

Invasion of HT1080s in A/2 invasion assays in the presence of either LPA or S1P was abolished by adding 5 μM of either GM6001, TAPI-0, or TAPI-1 to the culture media (Fig. 9A, B and data not shown). Likewise, adding 5 μM of either GM6001, TAPI-0, or TAPI-1 to the culture media blocked LPA-induced A2058 melanoma invasion (data not shown) further corroborating that lipid-induced tumor cell invasion is an MMP-dependent event. However, synthetic inhibitors of MMPs such as GM6001 lack specificity and inhibit a broad range of metalloproteinases. To identify the predominant class of MMPs involved in lipid-induced invasion of HT1080 cells, recombinant TIMP-1, -2, -3, or -4 were added to A/2 invasion assays at 5 μg/ml (Fig. 9A) TIMPs exhibit specificity for differing classes of metalloproteinases [soluble MMPs versus membrane-type MMPs and the ADAM (adisintegrin and metalloproteinase)] family of metalloproteinases [52,53]. Therefore, we used this property of the TIMPs to identify the particular class(es) of proteinase(s) involved in lipid-induced invasion of HT1080 cells. TIMP-2, -3, and -4, which are molecules known to inhibit both soluble, ADAMs, and membrane-type MMPs, each blocked lipid-induced invasion of HT1080 cells (Fig. 9A, B). The addition of TIMP-1, a TIMP capable of inhibiting soluble but not membrane-type MMPs, had no effect on lipid-induced HT1080 invasion. Analogous findings with TIMPs were seen in time course experiments using the side-view invasion system (See additional file 7: LPA+TIMP-1 to view the time-lapse imaging of LPA-induced HT1080 invasion in the presence of TIMP-1 and additional file 8: LPA+TIMP-3 to view the time-lapse imaging of LPA-induced HT1080 invasion in the presence of TIMP-3). These results indicate a requirement for membrane-bound MMPs in lipid-induced HT1080 invasion of 3D collagen matrices.

**Figure 10:**
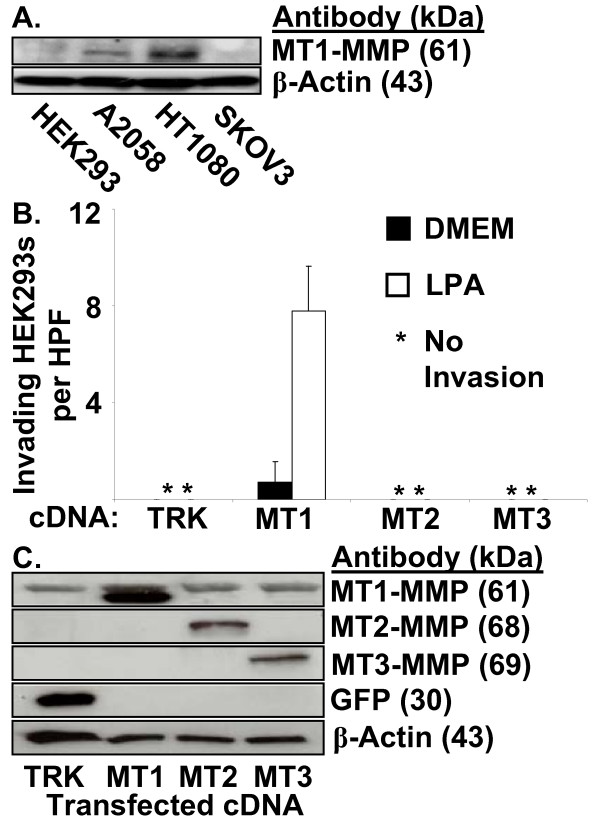
**HEK293 cells transfected with MT1-MMP cDNA invade 3D collagen gels in response to LPA**. (A) Tumor cell lysates were prepared for Western blot analysis. Lysates were probed for MT1-MMP to assess protein expression in the four tumor cell lines. Lysates were probed for Actin as a loading control. (B) HEK293 cells were transfected with the pAdTrack-CMV plasmid as a control, or plasmids encoding MT1-MMP, MT2-MMP, or MT3-MMP cDNA 24 hours prior to placement in invasion assays. Cells were allowed to invade 2.0 mg/ml collagen gels in the presence or absence of 1 μM LPA. Data are expressed as mean numbers of invading cells per HPF (20×) (± S.D.) from a minimum of 20 fields. (C) Lysates from HEK293 cells transfected with cDNAs encoding the designated genes were prepared for Western blot analysis and probed for GFP, MT1-MMP, MT2-MMP, MT3-MMP, or Actin as a loading control. TRK = pAdTrack-CMV, MT1 = MT1-MMP, MT2 = MT2-MMP, MT3 = MT3-MMP.

We next evaluated the intriguing observation that although SKOV3 and HEK293 cells markedly migrated in response to LPA in Boyden chamber assays, these cell lines were unable to invade 3D collagen matrices in response to these lipids as noted previously (Fig. 1 and Fig. 2B). Western blot analysis was performed to characterize endogenous MT1-MMP levels of the four tumor cell types (Fig. 10A). Remarkably, the level of MT1-MMP expression directly correlated with the ability of the tumor cell lines to invade 3D collagen matrices.

To investigate further the role of MT1-MMP in LPA-induced invasion, HEK293 cells (which do not invade in response to LPA) were transfected with MT1-MMP cDNA and allowed to invade in response to LPA for 48 hours. Once transfected, HEK293 cells invaded in the presence of LPA (Fig. 10B). Transfection with a control plasmid (pAdTrack-CMV), or plasmids encoding human MT2-MMP or MT3-MMP did not induce invasion despite appropriate expression of the various proteins (Fig. 10C). These data illustrate that MT1-MMP is required for LPA-induced invasion of HEK293 cells. The HEK293 cells also represent an interesting new model to assess tumor cell invasion due to their inability to invade unless genes such as MT1-MMP are supplied by transfection.

**Figure 11:**
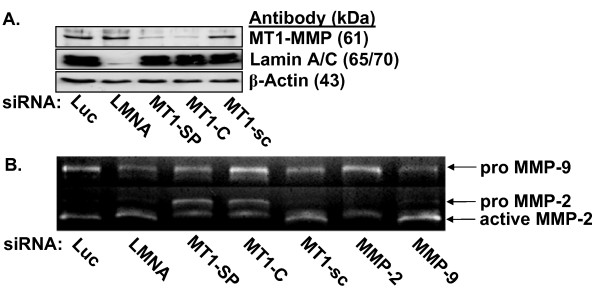
**siRNAs targeting MT1-MMP reduce protein levels and MMP-2 activity on gelatin zymography**. (A) Invading HT1080 cells lysates were prepared for Western blot analysis and probed for MT1-MMP and Lamin A/C to demonstrate siRNA efficiency and specificity. Actin was used as a loading control. (B) Conditioned media from siRNA-treated HT1080 cells were collected after 48 hours of invasion and gelatin zymography was performed. Arrows denote pro-MMP-9, and pro- and active forms of MMP-2.

**Figure 12:**
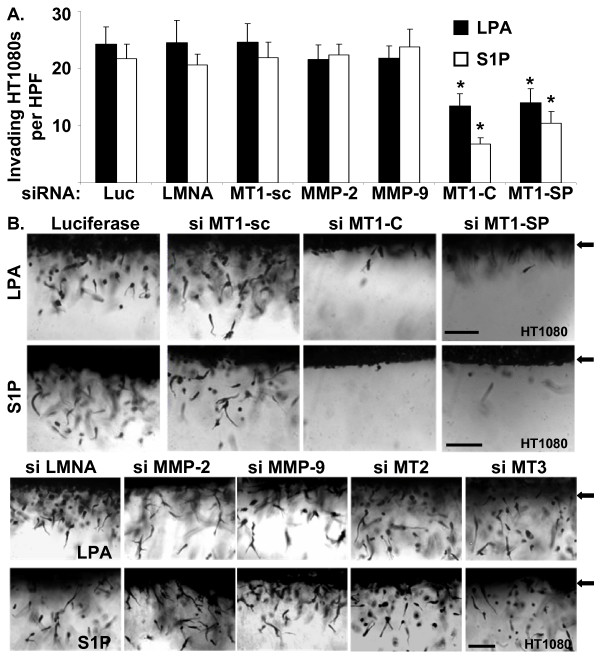
**MT1-MMP is required for lipid-induced invasion of HT1080 cells in 3D collagen gels**. (A) HT1080 cells were transfected with control siRNAs (Luciferase, Lamin A/C, or a scrambled MT1-MMP), siRNAs targeting soluble MMP-2 or MMP-9, or siRNAs targeting MT1-MMP (MT1-C, MT1-SP). Cells were allowed to invade 3D collagen gels in response to LPA or S1P for 48 hours prior to fixation and quantification of invasion as in Fig. 2. Data are expressed as mean numbers of invading cells per HPF (20×) (± S.D.) from a minimum of 20 fields per condition. Statistical significance of means was determined using t-test where * = p < 0.01 relative to Luciferase control. si = siRNA; Luc = Luciferase, LMNA = Lamin A/C, MT1-C = custom MT1-MMP, MT1-SP = *SMART*pool^® ^MT1-MMP, MT1-sc = scrambled MT1-MMP. (B) siRNA transfected HT1080 cells from panel A were imaged as in Fig. 2 to demonstrate tumor cell invasion. Magnification = 20×. Scale bars = 50 μm. Arrowheads indicate the position of the monolayer at initiation of invasion.

**Figure 13:**
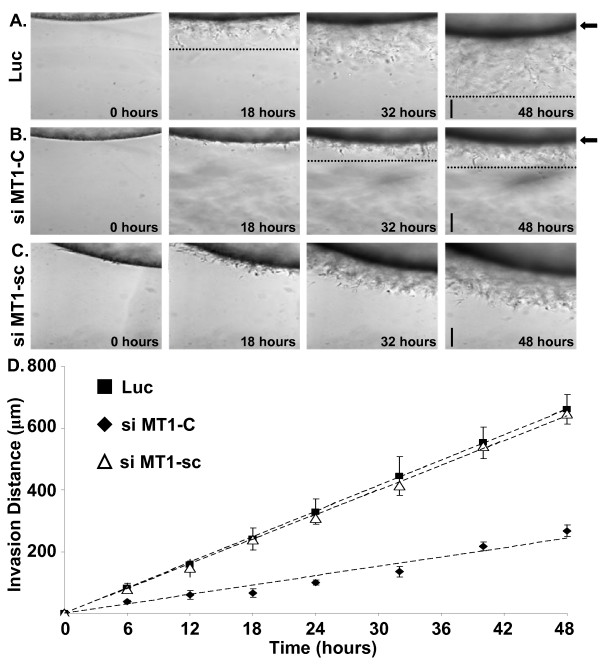
**The rate of HT1080 invasion is dependent on MT1-MMP**. HT1080 cells transfected with siRNA targeting Luciferase (A), MT1-MMP (custom) (B), or scrambled MT1-MMP (C) were seeded onto 3.75 mg/ml collagen gels in glass casings (see methods for details) and allowed to invade for 48 hours in the presence 1 μM LPA. Data were collected as in Fig. 3 and were plotted versus time (D). Rates of invasion were calculated using linear regression (see Table 1). Data are expressed as mean rate of invasion (n = 3 wells, ± S.D.) for each time point from triplicate experiments. Magnification = 10×. Scale bars = 200 μm. Dashed line indicates front of tumor cell invasion. si = siRNA; Luc = Luciferase, MT1-C = custom MT1-MMP, MT1-sc = scrambled MT1-MMP.

Next, we evaluated the role of MT1-MMP in lipid-induced invasion of HT1080 cells using the A/2 invasion assay. HT1080 cells were transfected with siRNAs targeting either the membrane-type MT1-, MT2-, or MT3-MMPs, soluble MMP-2 or MMP-9, or control siRNAs (scrambled MT1-MMP, Lamin A/C, or Luciferase GL2 duplex) prior to initiation of invasion assays. Transfection with either custom or *SMART*pool^® ^siRNAs targeting MT1-MMP were capable of inhibiting MT1-MMP protein expression on Western blot (Fig. 11A), pro-MMP-2 activation on gelatin zymography (Fig. 11B), and tumor cell invasion of 3D collagen matrices in response to either LPA or S1P (Fig. 12A, B). Transfection of HT1080 cells with siRNAs targeting the soluble MMPs (MMP-2 or MMP-9) or control siRNAs (Lamin A/C or Luciferase) had no influence on invasion (Fig. 12A, B). Similarly, invasion time course experiments revealed that HT1080 cells transfected with MT1-MMP siRNA exhibited nearly a 3-fold slower rate of invasion (~5 μm/hr vs. ~14 μm/hr for MT1-MMP and Luciferase, respectively) and decreased distance of invasion (~200 μm vs. ~700 μm) compared to Luciferase or scrambled MT1-MMP controls (Fig. 13A–D and Table 1). Collectively, our data identifies MT1-MMP as a key proteinase involved in lipid agonist-induced signaling and tumor cell invasion of 3D collagen matrices.

### MT1-MMP is required for lipid-induced invasion of tumor cells embedded within 3D collagen matrices

**Figure 14:**
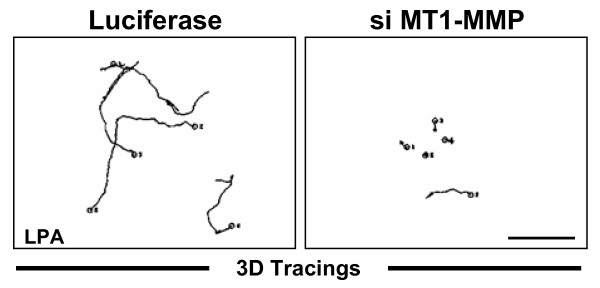
**MT1-MMP is required for tumor cell movement when cells are embedded within 3D collagen matrices**. Representative individual 24 hour tracings (illustrated in Fig. 7) of Luciferase or custom MT1-MMP siRNA transfected Nuc-GFP HT1080 cells embedded in 3D collagen matrices are presented (grayscale). Images were acquired every 10 minutes. Magnification = 20×. Scale bars = 100 μm.

Cells embedded within 3D collagen matrices can exhibit altered cellular behavior compared to cells cultured on the surface of collagen matrices [5,54,55]. Therefore, the role of MT1-MMP in lipid-induced invasion of HT1080 cells embedded within 3.75 mg/ml 3D collagen was assessed (as described in Figure 7). Nuc-GFP HT1080 cells were transfected with siRNAs targeting MT1-MMP (custom) or Luciferase (control) prior to initiation of the assays. Cell nuclei were tracked in 3D matrices as illustrated in Figure 7. Representative 24 hour tracings of nuclear movements are presented (Fig. 14). Cells transfected with custom MT1-MMP siRNA exhibited both decreased distance (~138 μm vs. ~90 μm – t-test p < 0.01) and velocity (~0.096 μm/min vs. ~0.064 μm/min – t-test p < 0.01) of invasion in 3D collagen matrices in the presence of LPA (Fig. 14 and Table 2) similar to that observed when HT1080 cells were treated with 5 μM GM6001 (Fig. 8 and Table 2). Additional experiments using 1 μM S1P yielded similar results (data not shown). These data indicate that lipid-induced dissemination of individual tumor cells embedded in 3D collagen matrices also requires MT1-MMP.

## Discussion

### LPA and S1P are collaborative regulators of tumor cell migration and invasion

LPA receptors are classic GPCRs, and have been shown to be abnormally expressed in cancer cells [25,27]. LPA is also elevated in the plasma of patients with ovarian carcinoma, indicating a link between LPA and early intraperitoneal dissemination [24,56]. Our data strongly indicate that LPA is a potent tumor cell motility factor when compared to other factors such as cytokines or chemokines. The recent discovery of autotaxin, an enzyme that generates LPA or S1P and is expressed by tumor cells, highlights the importance of LPA and S1P in tumor cell motility [22,57,58]. Intriguingly, our data suggest that LPA and S1P exert opposing effects on most tumor cell types (Fig. 1). Previous studies establish that lipid agonist signaling pathways may be particularly important in this context [21,27,59-62]. By identifying differential receptors involved in LPA and S1P signaling (i.e. LPA_1 _and S1P_1_) and the various downstream GTPase effectors (e.g. Rac1 and Cdc42), we hypothesize that motility and invasion are regulated by a balance between these pro- and anti-migratory signals. Contrary to other tumor cell lines, HT1080 cells are not inhibited by S1P; in fact, S1P actually stimulates HT1080 migration and invasion (Figs. 1 and 2). This cell line is also one of the most invasive tumor types known [41,42,63]. Thus, the loss of S1P inhibition, or the ability of S1P to stimulate a tumor cell, may directly contribute to metastatic potential. Studies investigating the molecular differences in lipid signaling and their relevance to tumor cell invasion and metastasis *in vivo *are underway [64] and should be the subject of future work.

### LPA and S1P regulate tumor cell invasion of 3D collagen matrices via MT1-MMP

When invasion of tumor cells was assessed, we found that an ability to migrate in response to LPA did not necessarily confer an invasive phenotype in 3D collagen matrices. In fact, only a small subset of tumor cell lines screened in our models show strong invasive behavior (i.e. HT1080 cells). By far the majority of cell lines do not substantially invade in 3D collagen matrices under the conditions described in this report (Fisher and Davis, unpublished observations). One possible explanation for this result is that Western blot analysis revealed that non-invasive cell types (HEK293 or SKOV3) expressed low levels of MT1-MMP, a proteinase known to be involved in tumor cell invasion (Fig. 10A) [5,6,37]. When HEK293 cells were transfected with cDNA encoding MT1-MMP, cells were able to invade collagen matrices containing LPA (Fig. 9B). Using HT1080 cells, we were able to again distinguish MT1-MMP as a critical molecule involved in lipid-induced invasion using siRNA transfection targeting this proteinase (Figs. 12, 13, 14) or the addition of chemical or protein inhibitors of this proteinase (Fig. 9). A critical question that remains unanswered is the mechanism by which LPA stimulates MT1-MMP-dependent invasion of collagen matrices. One possibility is that LPA is involved in localizing or stabilizing MT1-MMP to critical areas of the cell surface required for matrix proteolysis during 3D locomotion.

The work presented here shows that LPA can both stimulate tumor cell motility on 2D substrates (Figs. 1, 6, and 8) and tumor cell invasion of 3D collagen matrices (Figs. 2, 8, 10, and 12). Importantly, only the latter process depends on the MT1-MMP proteinase (Figs. 6, 8, 10, 14, and Table 2). Blockade of MT1-MMP using siRNA suppression or proteinase inhibitors strongly suppressed HT1080 movement in 3D collagen matrices and not on 2D collagen substrates. Also, HT1080 random cell motility on 2D substrates was not affected by LPA, while LPA clearly stimulated tumor cell movement/invasion in 3D collagen matrices. This data demonstrates that LPA strongly stimulates MT1-MMP-dependent tumor cell invasion behavior, and thus, is a critical co-factor along with matrix proteolysis in tumor cell invasion. An important unanswered question remains: how does LPA or S1P affect the ability of MT1-MMP to coordinate these invasive events? This central issue is fundamental to our understanding of how tumor and other cells such as endothelial cells invade 3D matrix environments. We hypothesize that LPA facilitates the localization of MT1-MMP to specialized areas of the cell membrane that are necessary to drive tumor cell locomotion through 3D matrices. Other possibilities include that LPA stimulates the co-association of MT1-MMP with critical co-regulatory molecules such as collagen-binding integrins (e.g. α2β1, α1β1, α10β1, α11β1). Preliminary experiments using siRNAs directed to MT1-MMP and the β1 integrin subunit show that suppressing both of these genes at the same time completely inhibits HT1080 invasion of 3D collagen matrices (Fisher and Davis, unpublished observations).

Previous studies have implicated other MMPs (i.e. MMP-2 and MMP-9) in tumor cell invasion and progression, and that LPA triggers upregulation or activation of MMP-2 [24,63,65-67]. However, in our system, siRNAs targeting MMP-2 or -9 or other MT-MMPs did not influence tumor invasion of collagen matrices (Fig. 12). Our data indicate that MT1-MMP is the predominant proteinase involved in lipid-induced invasion of collagen matrices. These conclusions are similar to a recent study showing the selective ability of MT-MMPs to stimulate tumor invasion with no evidence presented for a role of soluble MMPs [5]. Additionally, MT1-MMP acts upstream and can activate MMP-2. Thus, MT1-MMP appears to be the principle regulator of LPA-induced invasion. However, markedly decreasing levels of MT1-MMP via targeted siRNA transfection did not completely block completely invasion in HT1080 cells as effectively as the addition of GM6001 (Figs. 3, 8, and 12, 13, 14). In addition, the depth and numbers of invading MT1-MMP-transfected HEK293 cells were not as extensive as seen in HT1080 cells (Fig. 10). These results may indicate a role for additional molecules (e.g. collagen-binding integrins or multiprotein complexes involving MT1-MMP) in our invasion system. It is also plausible that other cell surface expressed metalloproteinases (i.e. other MT-MMPs or ADAMs) could contribute to degradation of matrix components and host/tumor interactions (i.e. tumor-driven angiogenesis) not addressed here [63]. Additional studies are needed to identify and address the role of key complementary molecules in tumor invasion and how this relates to lipid-agonist induced invasion.

## Conclusion

**Figure 15:**
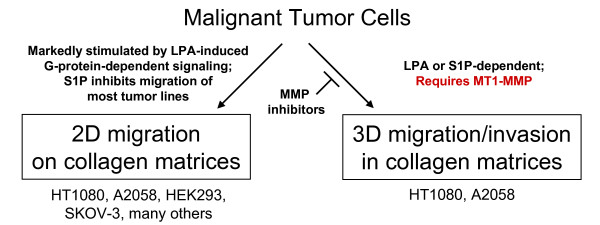
**Schematic diagram summarizing the roles of LPA, S1P, RhoGTPases, and MT1-MMP in tumor migration and invasion of collagen matrices**. LPA stimulates tumor cells migration while S1P inhibits migration of most tumor types on 2D matrix substrates. Lipid-induced invasion of 3D collagen matrices, but not migration, requires MT1-MMP. Only tumor cell invasion of 3D matrices is blocked by MMP inhibitors.

In this study, we demonstrated that LPA (and also S1P for HT1080s) stimulated the processes of motility and invasion through signaling of LPA_1_, S1P_1_, Rac1, Cdc42, and MT1-MMP. In most tumor cell lines, LPA markedly stimulated tumor cell motility while S1P functioned as an inhibitor of migration (except for HT1080 cells). In 3D collagen invasion assays, the invasion of HT1080 and A2058 cell lines mimicked their migration profile. HEK293 and SKOV3 cells did not invade 3D collagen matrices, despite a marked ability to migrate in response to LPA. The LPA_1 _and S1P_1 _GPCRs, along with Rac1 and Cdc42 were identified as crucial components of lipid agonist-induced invasion. Using our assays, we determined that LPA-induced tumor cell invasion of 3D collagen matrices required proteolysis distinguishing it from lipid-induced migration through porous membranes or on 2D substrata. MT1-MMP was identified as a key proteinase involved in lipid agonist-induced invasion in our systems. A schematic summarizing this data is presented in Figure 15. These results summarily address the importance of LPA and S1P in tumor motility and invasion, and directly link lipid agonist-induced tumor cell invasion of 3D collagen matrices to proteolysis via MT1-MMP.

It is clear that tumor cell migration and invasion are multifaceted events that require a myriad of cellular processes to act in concert to achieve cell movement and propulsion through natural barriers. The systems described here illustrate innovative ways to study the molecules involved in tumor locomotion. Using our systems, we identified molecular components (e.g. LPA_1_, S1P_1_, Rac1, Cdc42, and MT1-MMP) involved in lipid-induced HT1080 invasion (Figs. 4, 5, 6, 7, 8, 9, 10, 11, 12, 13, 14). We also quantitated the effects of LPA, S1P, or reduced MT1-MMP protein expression on the distance and rate of HT1080 invasion using a new time-lapse analysis system (Figs. 3, 8, 13, 14, and Tables 1 and 2). We believe that these *in vitro *3D microassay collagen invasion systems represent an important and attractive approach to study the molecular requirements for tumor invasion and the mechanisms underlying the ability of single tumor cells to invade through 3D collagen matrices. Identifying the key molecules involved in tumor migration and invasion will provide better comprehension of these processes and ultimately, may yield more specific cancer therapeutics, particularly those that target lipid agonist-induced migration and MT1-MMP-mediated invasion.

## Methods

### Reagents

48-well micro-chemotaxis chambers and polycarbonate membranes (8 μm pores) were purchased from Neuro Probe, Inc. (Gaithersburg, MD). D-Erythro-sphingosine 1-phosphate (S1P) and lysophosphatidic acid (LPA) 1-oleoyl-2-hydroxy-SN-glycero-3-phosphate, sodium salt were purchased from Avanti Polar Lipids (Alabaster, AL). Rat tail collagen type I was prepared at 7.1 mg/ml using the method previously described [68]. Tumor cell lines were purchased from American Type Culture Collection (ATCC) (Manassas, VA). Dulbecco's Modified Eagle Medium (DMEM), Lipofectamine 2000, and fetal bovine serum (FBS) were from Invitrogen Life Technologies (Rockville, MD). Bovine TIMP-1 and TIMP-2 were obtained from Chemicon Corp (Temecula, CA). Recombinant TIMP-3 and TIMP-4 were from R&D Systems (Minneapolis, MN). A rabbit polyclonal antibody was prepared against recombinant green fluorescent protein (GFP) as described [69]. Human MT1-MMP (AF918, R&D Systems, Minneapolis, MN), MT3-MMP (RP1-MMP-16, Triple Point Biologics, Forest Grove, OR), and Rac1 (ARC01, Cytoskeleton, Denver, CO), and monoclonal antibodies directed against human MT2-MMP (MAB916, R&D Systems), Lamin A/C (MAB3211, Chemicon Corp), Actin (JLA-20, Calbiochem, San Diego, CA), RhoA (ARH01, Cytoskeleton), and Cdc42 (610929, BD Tansduction, San Jose, CA) were used for Western blot analysis as described previously [70]. si*GENOME SMART*pool^® ^reagents were products of Dharmacon (Lafayette, CO). Plasmids encoding human MT1-MMP (EX-M0327-M02) and MT3-MMP (EX-Q0300-M02) were obtained from Genecopoeia (Germantown, MD). A plasmid encoding human MT2-MMP (TC118648) was purchased from Origene (Rockville, MD). Other reagents were obtained from Sigma (St. Louis, MO) unless noted.

### Cell culture

Tumor cells were cultured in T-75 flasks and supplied with DMEM supplemented with 10% FBS containing 100 μg/ml gentamicin (Invitrogen), 100 μg/ml of streptomycin, 100 Units/ml of penicillin, and 0.25 μg/ml Amphotericin B [Fungizone^®^, Invitrogen, Carlsbad, CA), and incubated at 37°C with 5.0% CO_2_. The stable HT1080 cell line expressing nuclear-GFP was cultured in media containing 10 μg/ml Blasticidin (Invitrogen). HEK293 cells were cultured on flasks pre-coated with 20 μg/ml type I collagen in PBS. Prior to use in experiments, cells were washed with phosphate buffered saline (PBS), removed from monolayer with trypsin-EDTA, neutralized with fetal bovine serum, and centrifuged. Cells were then washed in two, 10 ml volumes of DMEM to remove residual serum.

### Generation of stable HT1080s expressing nuclear-localized GFP

A vector expressing nuclear-localized GFP (Nuc-GFP) (vector pACGFP1-Nuc, 632431) was obtained from BD Biosciences-Clontech (Mountain View, CA). The Nuc-GFP insert was amplified via PCR using the following primers: Nuc-GFP UP: 5'-CACCATGGTGAGCAAGGGCGCCGAG-3' and Nuc-GFP DN: 5'-AGCAGTTATCTAGATCCGGTGGATC-3'. PCR product size was verified via agarose gel electrophoresis, and following PCR purification (Qiagen, Valencia, CA), was TOPO cloned into the pLenti6/V5-D-TOPO lentiviral vector (Invitrogen). Following TOPO cloning, One Shot Stbl3 Chemically Competent *E. coli *were transformed with DNA. Individual clones containing the Nuc-GFP insert were verified via PCR and sequence analysis. The 293FT lentiviral packaging cell line was obtained from the ViraPower Lentiviral Gateway Expression Kit (Invitrogen) and were cultured in 500 μg/ml G418. Nuc-GFP lentivirus was produced following the Invitrogen lentivirus protocol. HT1080s were infected with Nuc-GFP lentivirus and were incubated for 72 hours prior selection using 10 μg/ml of Blasticidin. HT1080s were cultured in Blasticidin for 10 days to ensure stable cell line selection, and Nuc-GFP expression was verified via fluorescent microscopy.

### 96-well A/2 vertical collagen invasion assay

Three-dimensional (3D) collagen type I invasion assays were adapted from previously described work [28]. Briefly, collagen gels (n = 3 gels per condition, 25 μl volume) were prepared as described [71] at a final concentration of 2.0 or 3.75 mg/ml and added to A/2 wells (4.5 mm diameter) of 96-well plates (Costar, Corning, NY). 1 μM S1P and/or LPA were incorporated into each gel prior to polymerization and equilibration at 37°C and 5% CO_2 _for 45 minutes. After equilibration, 100 μl of media containing a 1:250 dilution of RSII(+) (Transferrin, BSA, oleic acid, and insulin) and 35,000 tumor cells were added to each well. Cells were allowed to invade 3D collagen gels for 48 hours. Culture media was removed and collagen gels containing invading tumor cells were fixed in 3% glutaraldehyde in PBS for 30 minutes. Gels were stained with 0.1% toluidine blue in 30% methanol for 30 minutes prior to destaining with water. Transected gels were imaged using an inverted Nikon Eclipse TE2000-U microscope, camera, and Metamorph^® ^software (Molecular Devices Corporation, Downington, PA) or were acquired using an inverted Olympus microscope, camera, and Kodachrome 64T color film. Kodachrome images were scanned using a Polaroid SprintScan 4000 and PolaColor Insight Software (Polaroid, Cambridge, MA). To quantitate invasion of tumor cells in 3D collagen gels, cells were counted with eyepiece equipped with an ocular grid. For each condition, twenty random fields were selected and the number of invading cells per high power field (HPF) was counted manually at 20× magnification. Data are reported as mean number of invading cells per HPF (± S.D.).

### Effects of metalloproteinase inhibitors and pertussis toxin on migration or invasion

GM6001 (Calbiochem) and TAPI-1 (Chemicon) were reconstituted in DMSO at 2.5 mM. TAPI-0 (Chemicon) was reconstituted in DMSO at 0.25 mM. TIMP-1, TIMP-2, TIMP-3, and TIMP-4 were prepared at 100 μg/ml in 0.1% BSA. To determine the effects of MMP inhibitors on tumor cell migration and invasion, GM6001 (5 μM), TAPI-1 (5 μM), TAPI-0 (5 μM), and TIMPs 1–4 (5 μg/ml) were added to upper chambers of migration assays and directly to cell media in invasion assays. Likewise, 100 ng/μl of pertussis toxin was added to cell media where noted. Migration and invasion assays were performed as described.

### Transfection of HT1080 cells with siRNA

si*GENOME SMART*pool^® ^human MMP-2, MMP-9, MT1-MMP, MT2-MMP, MT3-MMP, LPA_1–3_, S1P_1–3_, RhoA, Rac1, and Cdc42 were purchased from Dharmacon. A 21-nucleotide custom MT1-MMP siRNA described previously by Weiss and colleagues [5] was prepared by Dharmacon. A Lamin A/C si*GENOME SMART*pool^® ^siRNA and a Luciferase GL2 duplex siRNA oligo (Dharmacon) were utilized as controls. All siRNA transfections were performed as described previously [72]; however, Lipofectamine 2000 and 20% serum were utilized for transfection purposes. Cells were allowed to recover from siRNA transfection for 18 hours prior to initiation of experiments. Cells were allowed to invade for 48 hours as described above, after which conditioned media were collected and stored at -20°C. Invading tumor cells were extracted from collagen matrices and stored at -20°C as described previously [72]. The remaining wells were fixed, stained, quantitated, and imaged as described.

### Transfection of HEK293 cells with plasmids encoding human MT-MMPs

Plasmids encoding human MT1-, MT2-, and MT3-MMP cDNA were purified using the Qiagen plasmid MIDI kit (Valencia, CA). T-25 flasks were pre-coated with type I collagen as described above, and HEK293 cells were cultured to 80% confluency in 5 ml of culture media. On the day of transfection, 6 μg of MT1, MT2, or MT3-MMP plasmid DNA or the control vector pAdTrack-CMV encoding GFP [73], kindly provided by Dr. Bert Vogelstein (Johns Hopkins University College of Medicine, Baltimore, MD), was diluted into Opti-MEM I (Invitrogen) and mixed with Lipofectamine 2000 to a final volume of 1 ml. The plasmid/Lipofectamine 2000/Opti-MEM solution was added to the cells prior to overnight incubation. Cells were washed, trypsinized, and seeded onto 2.0 mg/ml collagen matrices as described above. Transfected HEK293 cells were allowed to invade for 48 hours prior to fixation, staining, and quantification as described above. For each plasmid, protein expression was verified via Western blot analysis.

### Real-Time Quantitative PCR (RTQ-PCR)

*LPA*_1 _and *S1P*_1 _mRNA were quantified using the Applied Biosystem apparatus 7500 Fast Real-Time PCR system. Briefly, total RNA was extracted from siRNA-treated (Luciferase, LPA_1_, and S1P_1_) HT1080 cells using the Totally RNA Isolation kit (Ambion, Austin, TX) according to the manufacturer's instructions. RNA (5 μg) was reversed transcribed using StrataScript First Strand Synthesis System (Stratagene, Cedar Creek, TX). RTQ-PCR was performed in a final volume of 20 μl containing 2 μl of a 1:5 dilution of each RNA template, 10 μl of a TaqMan^® ^Fast Universal PCR Master Mix, and 1 μl of a TaqMan^® ^Gene Expression Assays (Applied Biosystems, Foster City, CA). Primers were designed using PrimerExpress sofware (Applied Biosystems), and targeted sequences between Exon 1 and Exon 2 (*S1P*_1_), between Exon 2 and Exon 3 (*LPA*_1_), and an inventoried eukaryotic *18S rRNA *(internal control). Each sample was assayed using three replicates for each primer. The 18S rRNA-specific primer was used as an internal standard. The cycling conditions were as follows: one cycle at 95°C for 20 seconds, followed by 40 cycles of PCR amplification, each consisting of 95°C for 3 seconds and 60°C for 30 seconds. Δ*Ct *values were obtained by subtracting the *Ct *value of the housekeeping gene *18s rRNA *from the *Ct *value for each indicated gene. The ΔΔ*C*_*t *_relative expression method was used to normalize *LPA*_1 _and *S1P*_1 _to *18s rRNA *as a housekeeping gene (comparative *C*_*t *_method). Data are reported as mean expression values normalized to *18s RNA *(± S.D.) of three replicates per siRNA treatment.

### Gelatin zymography and Western blotting

Gelatin zymography was performed with SDS-PAGE gels containing 8.5% acrylamide and 1 mg/ml porcine gelatin. Conditioned media from the HT1080 siRNA transfected cells were prepared under non-reducing conditions. Gel electrophoresis was performed at 150V for 55 minutes. Zymograms were incubated for three 20 minute periods in 100 mls of 2% Triton X-100 in water, washed twice in distilled water, placed in 25 mM Tris-HCl (pH 7.5) containing 5 mM CaCl_2_, and incubated overnight at room temperature. The following morning, gels were stained with 0.1% Amido Black in 30% methanol and 10% acetic acid for 15 min and destained in 30% methanol and 10% acetic acid for 1 hour at room temperature. Western blot analysis was performed using 10% or 14% (for RhoA, Rac1, and Cdc42) SDS-PAGE gels as described previously [70] and probed with antibodies at the following concentrations: GFP, 0.1 μg/ml; MT1-MMP, 2 μg/ml; MT2-MMP, 1 μg/ml; MT3-MMP, 1 μg/ml; Lamin A/C, 2 μg/ml; Actin, 0.1 μg/ml; RhoA, 2 μg/ml; Rac1, 5 μg/ml; and Cdc42, 2 μg/ml

### Time-lapse measurements and imaging of HT1080 invasion

Hollow glass casings (3 × 3 × 8 mm) were designed by Wale Apparatus (Hellertown, PA) to accommodate a collagen gel and tumor cells during invasion. Collagen gels were prepared at a final concentration of 3.75 mg/ml. 1 μM of S1P and/or LPA was incorporated into each gel before filling each glass casing with ~20 μl of collagen. Gels were allowed to polymerize and equilibrate at 37°C with 5% CO_2 _for 45 minutes. After equilibration, 50 μl of clear DMEM (Invitrogen) containing 25,000 HT1080 cells was added to the upper portion of the glass casing above the collagen. Cells were allowed to attach for 30 minutes before the glass casings were secured in a horizontal plane within the well of a 48 well plate (Costar) with sterile silicon grease. Each well was then supplied with 500 μl of media containing a 1:250 dilution RSII (+). Cells were allowed to invade for 48 hours at 37°C with 5% CO_2_. Digital images were captured directly every ten minutes using an inverted Nikon Eclipse TE2000-U microscope, camera, and Metamorph^® ^software (Molecular Devices Corporation, Downington, PA). Using Metamorph^®^, images of individual fields of invading tumor cells were arranged in sequential order. Distance of HT1080 invasion was measured using digital pixel measurements and plotted against time using Microsoft Excel^®^. Rate of invasion was determined using linear regression statistical analysis. Data are reported as the mean distance in micrometers of invading cell fronts (± S.D.). Five measurements for each time point of duplicate or triplicate experiments were performed.

### Assessment of chemotactic migration

Modified Boyden chambers were utilized to assess lipid-induced chemotactic directional migration. Polycarbonate filters (8 μm pores) were pre-coated with 1 mg/ml type I collagen or gelatin in PBS. LPA and/or S1P in DMEM containing a 1:250 dilution of reduced serum II supplement [RSII(-)] (Transferrin, BSA, no oleic acid, and insulin) were added to lower wells. Upper wells contained a 1:250 dilution of RSII(-) and lower wells contained 20 μg/ml of fibronectin for gelatin-coated membranes. 5 × 10^4 ^tumor cells were added to upper chambers and allowed to migrate for 4 hours. Filters were removed and stained with 0.1% amido black in 30% methanol and 10% acetic acid. Membrane-adherent migrating cells were calculated using Scion^® ^image and Microsoft^® ^Excel. Migration data are represented mean number of migrating cells × 10^3 ^(± S.D.) and represent the results of quadruplicate experiments.

### Tracking Nuclear-GFP HT1080 cells on collagen-coated plastic (2D) or embedded within in collagen matrices (3D)

To observe the movement of HT1080 cells embedded in 3D collagen matrices, nuc-GFP HT1080s or siRNA treated (Luciferase or custom MT1-MMP) nuc-GFP HT1080s were mixed 1:1 with non-fluorescent HT1080s. Cells were then incorporated into a 3.75 mg/ml collagen matrix (*n *= 3 cultures per condition) containing 1 μM LPA. 15 μl of collagen and cell mixtures were added to square 384-well tissue culture plates (VWR, West Chester, PA), and allowed to equilibrate for 1 hour prior to addition of 60 μl of media containing a 1:250 dilution of RSII(+). 5 μM GM6001 was added to the media as noted. Cells were allowed to invade the surrounding matrix for 48 hours at 37°C with 5% CO_2_. Digital fluorescent images were captured directly with Metamorph^® ^as described above. By tracking fluorescent nuclei in chronological frames, Metamorph^® ^generated cell tracings of the movements of individual cells. For 2D tracings, nuclear-GFP HT1080 cells were seeded on 6 well plates pre-coated (30 minutes) with 50 μg/ml of type I collagen and cultured with 2 mls of media containing a 1:250 dilution of RSII (+). GM6001 was added as described. Images and cell tracings were generated by Metamorph^® ^as described.

## Competing interests

The authors declare that there are no competing interests.

## Authors' contributions

KEF drafted the manuscript, designed and performed all 3D and siRNA experiments, carried out data analysis, and helped conceive the manuscript. AP designed and performed the initial migration experiments. WK assisted in the initial migration experiments and their design. NJA assisted in the initial migration experiments as well as the data analysis. WBS created the nuclear GFP lentivirus, participated in the 2D motility analysis, and assisted in drafting the manuscript. GED conceived the study, participated in its design and execution, and helped conceive and draft the manuscript.

## Supplementary Material

Additional file 1 LPA-induced HT1080 invasion of a 3D collagen matrix over 24 hours. Time-lapse image analysis over 24 hours of HT1080 invasion through a 3.75 mg/ml three-dimensional collagen matrix containing 1 μM LPA. Click here for file

Additional file 2 LPA-induced HT1080 invasion of 3D collagen matrix in the presence of 5 μM GM6001 over 24 hours. Time-lapse image analysis over 24 hours of HT1080 invasion through a 3.75 mg/ml three-dimensional collagen matrix containing 1 μM LPA with 5 μM GM6001 supplied in the media. Click here for file

Additional file 3 LPA-induced migration of Nuc-GFP HT1080 cells over 24 hours seeded on plastic coated with 50 μg/ml of collagen. Fluorescent time-lapse image analysis over 24 hours of Nuc-GFP HT1080 cells seeded on plastic coated with 50 μg/ml of collagen treated with 1 μM LPA Click here for file

Additional file 4 LPA-induced migration of Nuc-GFP HT1080 cells over 24 hours seeded on plastic coated with 50 μg/ml of collagen in the presence of 5 μM GM6001. Fluorescent time-lapse image analysis over 24 hours of Nuc-GFP HT1080 cells seeded on plastic coated with 50 μg/ml of collagen treated with 1 μM LPA and 5 μM GM6001 Click here for file

Additional file 5 LPA-induced invasion of Nuc-GFP HT1080 cells embedded in a 3D collagen matrix over 24 hours. Fluorescent time-lapse image analysis over 24 hours of Nuc-GFP HT1080 cells embedded in a 3.75 mg/ml collagen matrix containing 1 μM LPA Click here for file

Additional file 6 LPA-induced invasion of Nuc-GFP HT1080 cells embedded in a 3D collagen matrix over 24 hours with 5 μM GM6001. Fluorescent time-lapse image analysis over 24 hours of Nuc-GFP HT1080 cells embedded in a 3.75 mg/ml collagen matrix containing 1 μM LPA with 5 μM GM6001 supplied in the media. Click here for file

Additional file 7 LPA-induced HT1080 invasion of 3D collagen matrix in the presence of 5 μg/ml of TIMP-1 over 24 hours. Time-lapse image analysis over 24 hours of HT1080 invasion through a 3.75 mg/ml three-dimensional collagen matrix containing 1 μM LPA with 5 μg/ml of TIMP-1 supplied in the media. Click here for file

Additional file 8 LPA-induced HT1080 invasion of 3D collagen matrix in the presence of 5 μg/ml of TIMP-3 over 24 hours. Time-lapse image analysis over 24 hours of HT1080 invasion through a 3.75 mg/ml three-dimensional collagen matrix containing 1 μM LPA with 5 μg/ml of TIMP-3 supplied in the media. Click here for file
